# Multi-Environment Quantitative Trait Loci Mapping for Grain Iron and Zinc Content Using Bi-parental Recombinant Inbred Line Mapping Population in Pearl Millet

**DOI:** 10.3389/fpls.2021.659789

**Published:** 2021-05-18

**Authors:** Tripti Singhal, C. Tara Satyavathi, S. P. Singh, Aruna Kumar, S. Mukesh Sankar, C. Bhardwaj, M. Mallik, Jayant Bhat, N. Anuradha, Nirupma Singh

**Affiliations:** ^1^ICAR-Indian Agricultural Research Institute, New Delhi, India; ^2^ICAR-All India Coordinated Research Project on Pearl Millet, Jodhpur, India; ^3^Amity Institute of Biotechnology, Amity University, Noida, India; ^4^Regional Research Centre, ICAR-Indian Agricultural Research Institute, Dharwad, India; ^5^Acharya N. G. Ranga Agricultural University, Vizianagaram, India

**Keywords:** pearl millet, QTL mapping, iron and zinc content, SSR, RILs

## Abstract

Pearl millet is a climate-resilient, nutritious crop with low input requirements that could provide economic returns in marginal agro-ecologies. In this study, we report quantitative trait loci (QTLs) for iron (Fe) and zinc (Zn) content from three distinct production environments. We generated a genetic linkage map using 210 F_6_ recombinant inbred line (RIL) population derived from the (PPMI 683 × PPMI 627) cross using genome-wide simple sequence repeats (SSRs). The molecular linkage map (seven linkage groups) of 151 loci was 3,273.1 cM length (Kosambi). The content of grain Fe in the RIL population ranged between 36 and 114 mg/Kg, and that of Zn from 20 to 106 mg/Kg across the 3 years (2014–2016) at over the three locations (Delhi, Dharwad, and Jodhpur). QTL analysis revealed a total of 22 QTLs for grain Fe and Zn, of which 14 were for Fe and eight were for Zn on three consecutive years at all locations. The observed phenotypic variance (*R*^2^) explained by different QTLs for grain Fe and Zn content ranged from 2.85 (*QGFe.E3.2014–2016_Q3*) to 19.66% (*QGFe.E1.2014–2016_Q3*) and from 2.93 (*QGZn.E3.2014–2016_Q3*) to 25. 95% (*QGZn.E1.2014–2016_Q1*), respectively. Two constitutive expressing QTLs for both Fe and Zn co-mapped in this population, one on LG 2 and second one on LG 3. Inside the QTLs candidate genes such as Ferritin gene, Al^3+^ Transporter, K^+^ Transporters, Zn^2+^ transporters and Mg^2+^ transporters were identified using bioinformatics approaches. The identified QTLs and candidate genes could be useful in pearl millet population improvement programs, seed, restorer parents, and marker-assisted selection programs.

## Introduction

Pearl millet [*Pennisetum glaucum* (L). R. Br.] is a climate-resilient and health-promoting nutritious crop of the semi-arid tropics of Africa and Asia (Satyavathi et al., 2015). It is a staple food crop for approximately 90 million people that live in Asia and Africa and practice low-input subsistence farming and livestock production systems (Gangashetty et al., 2016). Pearl millet is an ideal crop in the context of global climate change due to its inherent tolerance to heat stress, salinity, and drought (Shivhare and Lata, 2016). Deficiencies or imbalanced intakes of energy or nutrients, particularly vitamins and minerals, cause a number of dysfunctions and diseases in humans, that are collectively referred to as “micronutrient malnutrition or hidden hunger.” Currently more than 2 billion people in the world are in risk of micronutrient malnutrition (Athar et al., 2020). Iron (Fe) and Zinc (Zn) deficiency in humans is caused by lack of purchasing power for highly nutritious food, reduced dietary intake and less bioavailability and bioutilization, especially among resource poor women, infants, and children in the developing countries (Shariatipour and Heidari, 2020). Nutrition supplementation, dietary diversification, and food fortification are among the targeted strategies available to address micronutrient deficiencies. Among, the above strategies, improving the nutrient profile of agricultural crops through genetic means is more pleasing as it is a one-time investment and becomes self-sustaining over a long period. Breeding of pearl millet varieties/hybrids with improved nutritional quality is one of the priority areas for providing nutritional security in the developing countries. Many breeders (Govindaraj et al., 2011, 2016; Rai et al., 2015; Satyavathi et al., 2015; Kumar et al., 2016) have identified huge variability for the trait and utilizing them in development of a handful of varieties and hybrids rich in micronutrients, which now playing a major role in nutritional security in dry lands. However, hybrid released so far in India is having as mean Fe content at par or less than HarvestPlus target level of 77 mg kg^–1^ (HarvestPlus, 2014). Underutilization of germplasm material with higher nutritional value may attribute due to poor agronomic qualities. Hence there is a great need to make constant effort to breed more and more parental lines which can combine both for yield and micronutrient content along with resistance/tolerance to drought, downy mildew and blast diseases, good keeping quality, etc., and showing a stable performance under different agro-geographical regions.

Genetic maps provide important information for detailed genetic analysis of quantitative traits and have been proven to be significant method for crop improvement (Mohan et al., 1997; Doerge, 2002). Identifying molecular markers which are closely associated with the nutritional trait such as Fe and Zn enable rapid introgression of such traits into elite backgrounds. Among the different marker systems, simple sequence repeat (SSRs) is considered as a powerful and practically useful marker system for marker-assisted breeding (MAB) because of its cheap and more user-friendly along with co-dominant nature, abundance, reproducibility, and variability (Wang et al., 2018). In addition, the size and type of the mapping population determine the gene effect for an economically important trait (Kumar et al., 2018). Recombinant Inbred Lines (RILs) are the best choice among different types of mapping population as they are homozygous as well as have undergone multiple cycles of recombination which helps in mapping of tightly linked markers. Further use of codominant markers along with large RIL population helps in the construction accurate high-resolution map.

There are so many examples that SSR markers being employed for marker assisted biofortification of crop varieties such as QPM and pro-vitamin rich maize hybrids (Gupta et al., 2009; Muthusamy et al., 2014) and development of high yielding basmati varieties with disease resistance (Singh et al., 2012).

There are two different strategies that the plant can able to uptake iron from the rhizosphere: Strategy I and Strategy II (Connorton et al., 2017). Most of the cereals like rice, maize, and wheat follow Strategy II through secretion of phytosiderophores (PSs) (Roberts et al., 2004). *TOM1* (Transporter of Mugineic acid family phytosiderophores)/*ZIFL4* is a member of major facilitator superfamily (MFS) which exports Fe^3+^-PS chelates in rice and barley (Nozoye et al., 2011). In addition to Strategy II, rice and barley both have a functional Iron-Regulated Transporter 1 (IRT1) homolog that allows direct Fe^2+^ uptake from the rhizosphere. Metal transporters, such as the P1B-ATPase family, zinc-regulated transporter (ZRT), iron-regulated transporter (IRT)-like protein (ZIP), natural resistance-associated macrophage protein (NRAMP) family, and cation diffusion facilitator (CDF) family, have been reported in plants, where these two metals play critical roles (Colangelo and Guerinot, 2006). Several genes/gene families involved in Fe and Zn homeostasis such as yellow stripe-like (YSL), ZRT, ZIP, NRAMP, nicotianamine synthase (NAS), nicotianamine aminotransferase (NAAT), heavy metal ATPases (HMA), zinc-induced facilitator-like (ZIFL), metal tolerant protein (MTP), and others have been characterized in cereal crops such as rice (Anuradha et al., 2012), sorghum (Kotla et al., 2019). In pearl millet, *PglYSL*, *PglZIP*, and *PglNRAMP* and a single gene for *PglFER* and *PglNAS* were identified (Mahendrakar et al., 2020). Hence, the aim of this study was to identify the QTL associated with grain iron and zinc content through construct of linkage map using SSRs markers in a large-sized RIL mapping population.

## Materials and Methods

### Plant Materials

The experiment materials comprised of 210 F_6:7_ RILs mapping population derived from an intra specific cross between PPMI 683 (P_1_, Female) and PPMI 627 (P_2_, Male) made during rainy season, 2009. The RIL population advanced by single seed descent method from F_2_ to F_6_ from single plants selected during F_2_, and was segregating for grain Fe and Zn contents, respectively. Parent (P_1_), i.e., PPMI 683 having high grain iron and zinc content and parent (P_2_), i.e., PPMI 627 having low grain iron and zinc content with checks (ICMB 98222 for high grain Fe and Zn content, ICMB 92111 for low grain Fe and Zn content) (Table 1). Both checks were planted after every 20^th^ row of the population.

**TABLE 1:** Pedigree of the parental lines used to develop F_6:7_ mapping populations along with the high and low iron checks.

**Parent**	**Iron (mg/kg)**	**Zinc (mg/kg)**	**Pedigree and developer**
PPMI 683	103	72	High iron rich restorer line developed from in-iadi germplasm resistant for downy mildew at IARI, New Delhi
PPMI 627	49	28	Promising medium maturity restorer derived through selection in Pusa composite 334 at IARI, New Delhi
ICMB 98222 (high Fe check)	85	45.8	ARD-288-1-10-1-2(RM)-5, developed by ICRISAT, India
ICMB 92111 (low Fe Check)	43.6	27.5	(81B × 843B)-11-1-1-B, developed by ICRISAT, India

The average grain yield of five randomly selected plants was taken as grain yield per plant and weight of randomly counted 1,000 seeds in each genotype was taken as 1,000 Seed Weight. This study is mainly focused on grain Fe and Zn content. Hence, only the two most important traits *viz*., seed yield per plant and 1,000 seed weight were considered for the study.

### Environments

A total of 210 RILs along with both parents and checks were evaluated in an alpha lattice design with two replicates (Patterson and Williams, 1976), under field conditions at three different geographical areas, showing all three pearl millet growing agro-climatic zones of India, (i) E1: ICAR–Indian Agricultural Research Institute, New Delhi (28°382′N, 77°802′E) representing Zone A with annual rainfall of more than 400 mm, (ii) E2: ICAR-IARI Regional Centre farm, Dharwad (15°212′N, 75°052′E) from zone B (covering the southern peninsular India), and (iii) E3: Agricultural Research Station, All India coordinated pearl millet project, Mandor, Jodhpur (26°252′N, 72°992′E) falling in zone A_1_ with annual rainfall <400 mm. The trials were conducted at all locations during three consecutive (south-west monsoon season, 2014, 2015, and 2016) growing seasons. Each RIL was sown on a four-rows plot of 4 m long with an inter-row spacing of 65 cm and plant-to-plant spacing of 10 cm. For a normal, healthy crop, standard pearl millet cultivation practices were strictly followed. Table 2 shows the weather and soil parameters at each location throughout the year. All climatic parameters except rainfall are presented as means over the crop-growing season (June–October). Rainfall is cumulative rainfall received during the crop-growing season. The relative humidity was calculated as the average of measurements taken in every morning and afternoon on each day.

**TABLE 2:** Geographical location, climatic, and edaphic factors over different years and locations.

**Environment parameter**	**New Delhi (E1)**			**Dharwad (E2)**			**Jodhpur (E3)**		
	**2014**	**2015**	**2016**	**2014**	**2015**	**2016**	**2014**	**2015**	**2016**
**Geographical identity**									
Latitude	28.70°N	28.70°N	28.70°N	15.45°N	15.45°N	15.45°N	26.35°N	26.35°N	26.35°N
Longitude	77.10°E	77.10°E	77.10°E	75.00°E	75.00°E	75.00°E	73.04°E	73.04°E	73.04°E
Altitude (m.s.l.)	219 m	219 m	219 m	751 m	751 m	751 m	231 m	231 m	231 m
**Climatic**									
Temp (max.)	34.98°C	34.48°C	33.88°C	27.8°C	29.13°C	26.77°C	34.4°C	36.8°C	33.08°C
Temp (min.)	24.08°C	23.58°C	22.38°C	20.47°C	20.76°C	20.47°C	25°C	25.6°C	24.76°C
Relative humidity (%)	70.2	70.7	75.2	80.9	78.06	82.81	63.5	60.3	62.28
Rainfall (mm)	472.6	717.3	1146.7	544.9	293.2	385.4	389	288	427
**Soil factors**									
Soil pH	7.89	8.1	7.87	7.1	6.8	6.9	8.4	8.2	8.2
Electrical conductivity (dSm^−1^)	0.23	0.25	0.22	0.18	0.2	0.2	0.09	0.1	0.08
% Organic content	0.26	0.24	0.25	0.31	0.29	0.3	0.22	0.21	0.27
Av. Fe (mg kg^–1^)	18.14	21.04	17.89	27.6	30.7	28.1	6.4	4.9	2.27
Av. Zn (mg kg^–1^)	2.3	4.62	2.12	1.7	2	1.9	3.2	2.9	1.35
Soil texture	Sandy loam	Sandy loam	Sandy loam	Silty clay	Silty clay	Silty clay	Loamy sand	Loamy sand	Loamy sand

### Sample Preparation and Extraction of Mineral Elements

At physiological maturity, panicles from five plants per plot were randomly selected from the central two rows and harvested separately and threshed using wooden mallet. Seeds from individual plants within the plot were mixed and washed with deionized double-distilled water to remove debris and other possible contaminations in order to obtain a representative sample for trait measurements. The grain samples were sealed in individual paper bags and placed in an oven at 75°C overnight. About 15 g of air-dried sample from each genotype was further used. Mineral (Fe and Zn) content in the grains during initial year (2014) was measured with EDXRF instrument (a non-destructive, bench-top, energy-dispersive X-ray fluorescence spectrometry, Oxford Instruments X-Supreme 8000) at all three locations (Anuradha et al., 2017). Later, in 2015–2016, a more precise method, di-acid digestion of ground floor samples was used (Dhyan et al., 2005), followed by readings taken on an atomic absorption spectrometer (AAS, ZEE nit 700 tech Analytikjena). To determine whether higher grain mineral content is due to contamination with dust, soil, or is inherent, ICPMS (Nex ION 300X, Perkin Elmer, United States) was used to analyze genotypes having high iron content (>90 mg kg^–1^) (Pfeiffer and McClafferty, 2007).

### Statistical Analysis

Descriptive statistics and Analysis of Variance (ANOVA) was carried out using PBTools v1.4. Histograms and correlations between pairs of traits were estimated through Pearson correlation co-efficient using R software. The model used for ANOVA was:

$$z_{ijklm}=\mu +y_{i}+e_{j}+y_{eij}+r_{ijk}+b_{ijkl}+g_{m}$$

+(yg)im+(eg)jm+(yeg)ijm+Σijklm′′

Where μ is the grand mean; *yi* is the fixed effect of year *i*; *ej* is the fixed effect of location *j*; *ye*_*ij*_ is the fixed effect of interaction between year *i* and location *j*; *gm* is the random effect of genotype *m* and is ∼NID (0, σ^2^*g*); *r*_*ijk*_ is the random effect of replication in location *j* and year *i* and is ∼NID (0, σ^2^*r*); *b*_*ijk*__*l*_ is the random effect of block l nested with replication *k* in location *j* and year *i* and is ∼NID (0, σ^2^_*b*_); (*yg*)_*im*_ is the random effect of the interaction between genotype *m* and year *i* and is ∼NID (0, σ^2^_*yg*_); (*eg*)_*jm*_ is the random effect of the interaction between accession *m* in location *j* and ∼NID (0, 0, σ^2^_*eg*_); (*yeg*)_*ijm*_ is the random effect of the interaction effect of the genotype *m* in year *i* and location *j* and ∼NID (0, 0, σ^2^_*yg*_); and *ε_*ijklm*_* is the random residual effect and ∼NID (0, 0, σ^2^_ε_).

Analysis of variance was also conducted using data from each environment for grain Fe and Zn content.

Heritability (*H*^2^, broad sense) at individual environment was estimated from analysis of variance. The formula used was-

$$H^{2}=\frac{\sigma_{g}^{2}}{\sigma_{g}^{2}+\frac{\sigma_{\varepsilon }^{2}}{r}}$$

Whereas Heritability (*H*^2^) estimates across environments were estimated by the formula-

$$H^{2}=\frac{\sigma_{g}^{2}}{\sigma_{g}^{2}+\frac{\sigma_{yg}^{2}}{y}+\frac{\sigma_{eg}^{2}}{l}+\frac{\sigma_{yeg}^{2}}{yl}+\frac{\sigma_{\varepsilon }^{2}}{ryl}}$$

Where *r*, *y*, *l* denotes the number of replicates, years and environments, respectively.

The REML model also produced best linear unbiased predictors (BLUPs) of each genotype, thereby adjusting the influence of the neighboring rows. These BLUPs were used for downstream analysis.

### Genomic DNA Extraction and Genotyping

The genomic DNA was extracted from fresh and young leaf tissue of each RIL and the parents by using modified cetyltrimethyl-ammonium bromide (CTAB) method (Aboul-Maaty and Oraby, 2019). Polymorphism was assessed using 372 SSR markers, EST-SSRs (ICMP and IPES series), gSSRs (PSMP and CTM series), and gene-based markers. SSRs were amplified in 10 μl PCR reaction mixture containing 25–30 ng genomic DNA, 100 μM of each of the four dNTPs, 1.5 mM MgCl_2_, 5 pmol/μl of forward and reverse primer, 1 U of Taq DNA polymerase (Banglore-genei, Merck) and 1× PCR buffer in Biorad My Cycler Thermal Cycler, United States. PCR conditions were used as described by Anuradha et al. (2017). The amplified product was checked using 1.2% agarose gel and PCR products were separated by 10% polyacrylamide gel electrophoresis at 120 V (C.B.S. Scientific vertical electrophoresis unit). The SSR marker alleles were scored manually by comparing the position of bands with that of 100 bp ladder from bottom to top (smallest to largest). Based on the position, the allele size in base pairs (bp) was recorded for all polymorphic SSRs in all 210 RILs studied. The parent P_1_ (PPMI 683) was scored as “A” and parent P_2_ (PPMI 627) was scored as “B” and both type band present on single locus were designed as “H”. Diffused or ambiguous bands were recorded as missing bands with “0” notation. The chi-square method was conducted to test whether the inherited alleles of the mapping population were consistent with Mendelian segregation ratios. The segregation ratio across the mapping population was tested against a 1:1 ratio. The segregation pattern that did not fit either ratio (*p* < 0.05) were treated as distorted.

### Linkage Analysis and QTL Mapping

The linkage map was constructed using 151 polymorphic SSR markers. The markers that were not taken for the linkage analysis were those with: a minor allele frequency of less than 5%, more than 20% missing data, and those that were monomorphic between the parents. The MAPMAKER/EXP3.0 software was used to develop a genetic linkage map (Lander et al., 1987). The maximum recombination fraction (θ) was set at 0.49 and the critical logarithm of odds (LOD) score for the test of marker pair independence was set at 3. Markers with high segregation distortion (*p* ≤ 0.01) were removed in a preliminary analysis of the data. Subsequently, the linkage analysis of genotyping data for all the markers including those with distorted segregation was used. Using MapChart software, a graphical map was generated (Voorrips, 2002). The consensus map developed by Supriya et al. (2011) and Rajaram et al. (2013) was used as a reference map in the current study for grouping markers and constructing the map. QTL analysis was carried out using the Inclusive Composite Interval Mapping method (ICIM). The ICIM approach uses a strategy in which a stepwise regression is firstly performed, so markers with significant effect on QTL are selected. ICIM-ADD method was used with the multi-environmental model builtin QTL IciMapping (Li et al., 2015). Significant LOD thresholds were set at 5% tail of the null distribution in a 1,000 permutations test (Luciano Da Costa et al., 2012).

### Quantitative Trait Loci Validation

All QTL intervals identified for grain Fe and Zn content were analyzed *in-silico* with reference genome, Tift23D_2_B_1_–P_1_–P_5_ (PROJECT ID: PRJNA294988.) in order to navigate the presence of candidate gene associated with Fe and Zn content in grain. The physical position of flanked markers of identified QTLs were obtained from NCBI-BLASTn^1^. The sequence similarities searched against related cereals (*Setaria italica*) by Ab initio gene prediction method by using Fgenesh software for the identification of potential protein coding regions (Salamov and Solovyev, 2000)^2^. For each predicted exon similarity searches were run using the BLAST program on protein and EST databases (Altschul et al., 1997).

## Results

### Phenotypic Evaluations

**FIGURE 1:**
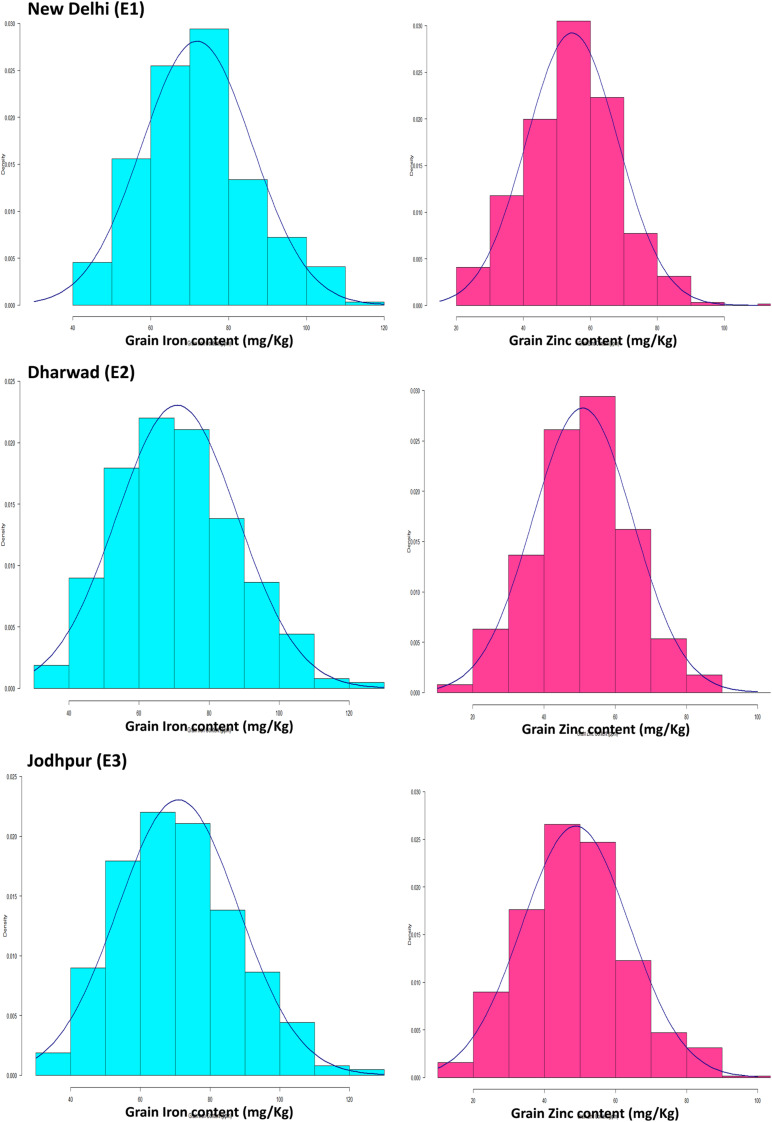
Histograms of grain Fe and Zn in mapping populations of recombinant inbred lines evaluated across the year (2014–2016) at Delhi, Dharwad, and Jodhpur location.

The mean performance of parents and range of trait values of RILs during three consecutive years (2014, 2015, and 2016) at E1, E2, and E3 are summarized in Table 3. Both traits (Fe and Zn) were approximately normally distributed when analyzed across the years at their respective locations (Figure 1). The levels of grain Fe in RILs ranged from 43.27 to 108.33 mg kg^–1^, 33.34–118.52 and 32.88–115.47 mg kg^–1^, while the range of grain Zn content was 22.4–94, 20.97–120.87, and 17.33–105.17 mg kg^–1^ across the years at E1, E2, and E3, respectively. The mean BLUP value of grain Fe and Zn content in both parents and RILs were higher across the described years at E1 when compared to E2 and E3, whereas mean BLUPs of grain Fe and Zn for both parents was lowest at E3 across the years. For grain Fe and Zn studied in different environments, both parents and RILs showed a wider range of variation.

**TABLE 3:** Grain Fe and Zn content (mg Kg^–1^) ±SE of the parents along with RILs of F_6:7_ mapping populations over 3 years and three diverse locations.

**Locations**	**Delhi (E1)**								**Dharwad (E2)**								**Jodhpur (E3)**							
**micron utrient**	**Iron**				**Zinc**				**Iron**				**Zinc**				**Iron**				**Zinc**			
**Years**	**14–15**	**15–16**	**16–17**	**Across year**	**14–15**	**15–16**	**16–17**	**Across year**	**14–15**	**15–16**	**16–17**	**Across year**	**14–15**	**15–16**	**16–17**	**Across year**	**14–15**	**15–16**	**16–17**	**Across year**	**14–15**	**15–16**	**16–17**	**Across year**
PPMI 683	98.5 ± 2.5	100.5 ± 2.5	98 ± 6	99 ± 0.76	83 ± 15	78.5 ± 3.5	91 ± 5	84.17 ± 3.66	95.56 ± 1.17	94.06 ± 1.33	96.56 ± 1.33	95.39 ± 0.73	70.37 ± 2	76.04 ± 2.67	63.54 ± 0.83	69.98 ± 3.61	83.95 ± 2.52	84.2 ± 1.2	82.2 ± 2.13	83.45 ± 0.63	58.6 ± 2.33	59.77 ± 2.67	64.27 ± 2	60.88 ± 1.73
PPMI 627	51 ± 1	53 ± 1	47 ± 1	50.33 ± 1.76	32 ± 0	33 ± 3	26.5 ± 0.5	30.5 ± 2.02	37.74 ± 1.67	35.07 ± 1.5	41.07 ± 2.17	37.96 ± 1.74	23.65 ± 2.5	21.15 ± 1.33	27.65 ± 1.83	24.15 ± 1.89	35 ± 0.8	34.33 ± 0.8	33.33 ± 0.93	34.22 ± 0.49	20 ± 2	18.5 ± 1.83	17.5 ± 2.33	18.67 ± 0.73
Pop (MEAN)	71.15 ± 1.01	71.45 ± 0.96	*71.55 ± 0.96*	71.38 ± 0.96	51.41 ± 1.01	55.31 ± 0.88	53.47 ± 0.93	53.39 ± 0.84	70.99 ± 1.18	70.92 ± 1.21	70.82 ± 1.21	70.91 ± 1.19	51.43 ± 0.89	49.67 ± 1.05	51.58 ± 1.02	50.9 ± 0.93	69.69 ± 1.03	69.78 ± 1.01	69.59 ± 1.05	69.69 ± 1.02	48.61 ± 1.05	49.82 ± 1.02	48.26 ± 1.1	48.9 ± 1.04

The seed yield per plant and 1,000 seed weight of the 210 RILs along with both parents varied greatly. At E1 during three consecutive years (2014–2016), 1,000 seed weight ranged from 6.75 to 12.17 g with a mean of 9.05 g, and seed yield per plant ranged from 14.09 to 36.21 g with the mean of 24.05 g. And at E2 from 2014 to 2016, 1,000 seed weight ranged from 6.77 to 12.33 g with a mean of 9.09 g, and seed yield per plant ranged from 19.05 to 35.37 g with a mean of 26.81 g. From 2014 to 2016, 1,000 seed weights in E3 ranged from 6.48 to 11.41 g, with an average of 8.38 g, and seed yield per plant was 18.20–39.8 g, with an average of 26.75 g.

The ANOVA indicated a highly significant G × *Y* interaction [*p* < 0.0001, *F*_(66.45)_ = 1.112 for Fe] and [*p* < 0.0001, *F*_(62.43)_ = 1.112 for Zn]. The pooled mean, range, coefficient of variation (CV), genotypic variance (σ^2^_*g*_), residual variance, broad sense heritability (*H*^2^), least square difference (LSD) and G × E variance in RILs evaluated during 2014–2016 cropping season at E1, E2, and E3 has shown in Table 4. In the RIL population, the genotypic components of variance (σ^2^_*g*_) for both traits were significant, and the operational heritability (*H*^2^) estimates ranged from 0.83 (Zn) to 0.88 (Fe) (Table 4). The high heritability shows that much of the phenotypic variance in the population is genetically controlled and QTL can be mapped with a high degree of reliability. Broad-sense heritability was usually high enough to allow effective QTL mapping, indicating that the traits studied have moderately high to high proportions of genetic variance. Correlation coefficients (*r*) for grain Fe and Zn content were found statistically significant (*p* < 0.001) for each year of evaluations for each location.

**TABLE 4:** Estimates of mean, variance components (2014, 2015, and 2016), broad-sense heritability and LSD at Delhi, Dharwad, and Jodhpur for grain Fe and Zn content in RILs.

**Traits**	**Environment**	**Mean**	**Range (mg kg^–1^)**	**CV %**	**Genotype variance**	**Residual variance**	**Heritability**	**LSD**	**G × E variance**
Fe									
1	New Delhi_2014	71.03 ± 1.00	41.4–111.5	4.47	211.25	10.08	0.88	4.66	70.00**
2	New Delhi_2015	72.71 ± 0.95	42.1–111	2.23	198.91	2.62	0.89	2.3	
3	New Delhi_2016	71.95 ± 0.96	44–108.5	2.44	203.28	3.09	0.89	2.69	
4	New Delhi across years	71.89 ± 0.96	43.27–108.33	3.19	161.09	5.27	0.81	7.51	
5	Dharwad_2014	70.95 ± 1.18	34.63–119.35	3.79	293.4	7.23	0.89	3.75	
6	Dharwad_2015	70.86 ± 1.21	17.71–125.7	3.99	311.4	8.01	0.89	3.94	
7	Dharwad_2016	70.81 ± 1.21	33.8–123.19	4.17	308.43	8.7	0.89	4.11	
8	Dharwad across years	70.87 ± 1.19	33.34–119.57	3.96	301.49	7.86	0.89	3.62	
9	Jodhpur_2014	69.6 ± 1.03	32.6–114.8	4	224.01	7.74	0.88	4.01	
10	Jodhpur_2015	69.68 ± 1.01	36.47–118	4.26	214.31	8.82	0.88	4.12	
11	Jodhpur_2016	69.48 ± 1.05	15.8–100	4.15	231.69	8.3	0.88	4	
12	Jodhpur across years	69.58 ± 1.02	32.88–115.47	4.02	223.56	7.84	0.89	2.94	
Zn									
13	New Delhi_2014	50.85 ± 1.01	20.1–114	5.01	200.13	6.48	0.88	3.88	107.00**
14	New Delhi_2015	58.79 ± 0.88	22.4–90	3.28	147.25	3.72	0.89	2.73	
15	New Delhi_2016	54.1 ± 0.94	21.2–89	3.18	188.94	2.96	0.89	2.64	
16	New Delhi across years	54.58 ± 0.84	22.4–94	3.84	110.81	4.38	0.83	8.72	
17	Dharwad_2014	51.39 ± 0.88	20.97–114.2	5.2	164.6	7.13	0.88	3.78	
18	Dharwad_2015	49.66 ± 1.04	17.71–125.7	5.71	230.7	8.04	0.88	3.94	
19	Dharwad_2016	51.52 ± 1.01	18.99–122.7	5.49	213.51	8	0.88	4	
20	Dharwad across years	50.86 ± 0.93	20.97–120.87	5.46	176.41	7.7	0.85	6.3	
21	Jodhpur_2014	48.52 ± 1.04	17.6–102	5.68	227.84	7.58	0.88	3.84	
22	Jodhpur_2015	49.72 ± 1.01	15.8–100	5.79	216.96	8.29	0.88	4	
23	Jodhpur_2016	48.19 ± 1.09	15–113.5	5.77	253.4	7.73	0.88	3.87	
24	Jodhpur across years	48.81 ± 1.03	17.33–105.17	5.74	224.59	7.84	0.88	4.32	

Significance at **p < 0.01.

Homogeneity of variance tests based on Bartlett’s test indicated homogeneous error variance for both the traits which allowed for a combined analysis across the years for all locations. When performing a combined ANOVA, the year was considered as a random and genotypes were considered as fixed effects. Combined ANOVA showed significant Genotype × Environment Interaction (GEI) (*p* < 0.001), exhibiting the influence of changes in environment on the grain micronutrient contents of genotypes. It was observed that Genotypes (G) and GEI effects were highly significant (*p* < 0.01) and the contribution of year is very less for both the traits (Table 5).

**TABLE 5:** Mean sum of square of Fe and Zn at all locations based on combined analysis over three years for RILs and parents.

**Sources**	**Degrees of freedom**	**Delhi_Fe**	**Delhi_Zn**	**Dharwad_Fe**	**Dharwad_Zn**	**Jodhpur_Fe**	**Jodhpur_Zn**
Genotypes	211	1,059.8**	806**	1,823**	1,120**	1,349.8**	1,372**
Environments (years)	2	300.7	6,755**	2	456	4.4	274
Block	3	2,175.9**	1,765**	7,044**	6,747**	6,652**	7,290**
Year × genotype	422	92.9**	141**	14**	61**	7.9	24**
IPCA 1	212	108.5**	148**	26**	108**	13.1**	37**
IPCA 2	210	77.1**	134**	2	14**	2.7	11**
Total	1271	215.3	198	328	227	246.6	257

*IPCA, interactive principal component analysis.*

Significance at **p < 0.01.

### Correlation of Grain Fe, Zn, 1,000 Seed Weight and Yield

Grain Fe and Zn content were highly (0.711) and significantly (*p* < 0.01) correlated (Table 6). Thousand seed weight was also observed to be positively correlated with seed yield per plant (0.579, *p* < 0.01). For successful incorporation of high levels of both grain Fe and Zn content, association between these micronutrients and their association with thousand seed weight and yield is important to plan a successful breeding approach so as to develop new improved lines or varieties having higher grain yield along with enhanced levels of grain micronutrients.

**TABLE 6:** Correlation of grain iron and zinc content with thousand seed weight and grain yield.

	**Fe**	**SYPP**	**TSW**	**Zn**
Fe	1	0.049	0.032	0.711**
SYPP		1	0.579**	0.059
TSW			1	0.059
Zn				1

Fe, SY, TSW, and Zn are Grain iron content in mg/kg, Seed yield per plant in g, thousand seed weight in g, and Grain zinc content in mg/kg.

**Significance at *p* < 0.01.

### Linkage Map Construction

**FIGURE 2:**
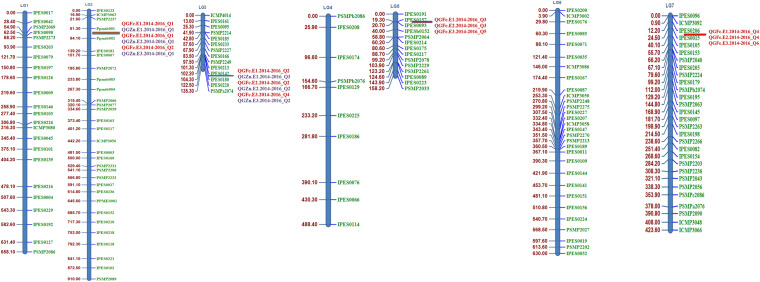
Linkage map (linkage groups LG 1–LG 7) of the pearl millet recombinant inbred line (RIL) population based on the cross (PPMI 683 × PPMI 627). QTL identified in red color font indicates the grain Fe QTLs and QTL identified in blue color font indicates the grain Zn QTLs.

For the polymorphism survey of both parental lines, 366 SSR primer pairs (IPES, PSMP, and ICMP series) and six gene-based primers were used. Of them, 151 (40.59%) SSRs detected polymorphism between the two parents. Polymorphic SSR markers included 94 IPES, 42 PSMP, and 11 ICMP series primer pairs and four newly synthesized primer pair named as Ppmsb (Pusa pearl millet SSR biofortification) (Figure 2). These polymorphic primers were further used for linkage map construction with LOD score 3.0.

The total length of the linkage map was 3273.1cM (Kosambi). The individual linkage group ranged from 910 cM for LG 2 with the highest number of markers (31) to 135.3 cM for LG 3 with 14 markers, while the least number of markers were assigned on LG4 (10) followed by LG 3 (14) and LG 5 (14). The average interval size was 8.79 cM. The average LG length was 467.5 cM with an average of 21.57 loci. The average adjacent-marker interval lengths ranged from 3.07 cM (LG 3) to 14.91 cM (LG 2) followed by 11.34 cM (LG 1). Map distance between adjacent markers varied from 0.9 to 49.2 cM (LG 1) and 8.33% of the intervals (31 out of 372 intervals) were smaller than 10 cM. Details of all linkage group, total markers, skewed marker and their percentage, total map length (cM) and average distance (cM) were given in Table 7.

**TABLE 7:** Details of the simple sequence repeats (SSRs)-based linkage map of the pearl millet RIL population based on the cross PPMI 683 × PPMI 627.

**Linkage group**	**SSR marker loci**	**Skewed SSRS**	**Total marker loci**	**Skewed loci %**	**Total length (cM)**	**Average distance (cM)**
1	23	35	58	60.34	658.1	11.34
2	31	30	61	49.18	910	14.91
3	14	30	44	68.18	135.3	3.075
4	10	30	40	75	361.8	9.045
5	14	35	49	71.42	158.2	3.22
6	30	30	60	50	626.1	10.43
7	29	31	60	51.6	423.6	7.06
Total	151	221	372	425.72	3,273.1	59.08
Average per linkage group	21.57	31.57	53.14	59.4	467.5	8.79

### Quantitative Trait Loci Analysis

The multi-environmental QTL analysis exhibited the association of various genomic regions with grain Fe and Zn content at all the three locations during three consecutive years, 2014, 2015, and 2016 (Table 8). The analysis indicated the presence of four significant common QTL for grain Fe content, flanked by following markers Ppmsb001–Ppmsb002, IPES0142–IPES0180, IPES0157–IPES0093, and IPES0206–IPES0015, which explained the total phenotypic variance of 18.34, 17.98, and 15.98% for QTL1 on LG 2, 14.99, 16.26, and 14.8% for QTL 2 on LG 3, 19.66, 18.83, and 16.85% for QTL 3 on LG 5, 15.66, 16.61, and 14.56% for QTL 4 on LG 7 across three consecutive years (2014–2016) at E1, E2, and E3, respectively. These four QTLs were named as: *QGFe.E1.2014–2016_Q1*, *QGFe.E1.2014–2016_Q2*, *QGFe.E1.2014–2016_Q3*, and *QGFe.E1.2014–2016_Q4* for Delhi location (E1), *QGFe.E2.2014–2016_Q1*, *QGFe.E2.2014–2016_Q2*, *QGFe.E2.2014–2016_Q3*, and *QGFe.E2.2014–2016_Q4* for Dharwad location (E2), and *QGFe.E3.2014–2016_Q1*, *QGFe.E3.2014–2016_Q2*, *QGFe.E3.2014–2016_Q3*, and *QGFe.E3.2014–2016_Q4* for Jodhpur location (E3) (Q = QTL; G = grain; Fe = iron). In addition, two more QTLs for GFe were also found at E3, flanked by IPES0101–IPES0139 and IPES0027–IPES0236 and having very less PVE of 2.92 and 2.85% on LG 1 and LG 2, respectively. The genome wide significant threshold was LOD = 3.5 for E1, E2, and E3.

**TABLE 8:** Quantitative trait loci for grain Fe (GFe) and Zn (GZn) content in RIL over three locations.

**QTL name**	**LG**	**Position (cM)**	**Left marker**	**Right marker**	**Intermarker distance (cM)**	**LOD**	**LOD**	**LOD**	**PVE**	**PVE**	**PVE**	**Add**	**AbyE_01**	**AbyE_02**	**AbyE_03**	**Left CI**	**RightCI**
															
							**(A)**	**(A by E)**		**(A)**	**(A by E)**						
*QGFe.E1.2014–2016_Q1 (QFe2.1)*	2	138.9	Ppmsb 001	Ppmsb 002	32.8	23.16	23.15	0	18.34	18.27	0.07	3.46	0.26	–0.26	0	135.9	141.9
*QGFe.E1.2014–2016_Q2 (QFe3.1)*	3	121	IPES0142	IPES 0180	2.1	19.11	19.1	0.01	14.99	14.97	0.01	3.13	0.08	–0.13	0.06	110	124
*QGFe.E1.2014–2016_Q3 (QFe5.1)*	5	21.3	IPES 0157	IPES 0093	1.4	24.92	24.69	0.23	19.66	19.58	0.09	3.58	–0.16	0.34	−0.18	19.3	30.3
*QGFe.E1.2014–2016_Q4 (QFe 7.1)*	7	24.9	IPES 0206	IPES 0015	12.3	20.06	20.03	0.02	15.66	15.61	0.05	3.21	0.25	–0.1	−0.14	21.9	27.9
*QGZn.E1.2014–2016_Q1 (QZn 2.1)*	2	138.9	Ppmsb 001	Ppmsb 002	32.8	21.39	20.85	0.54	25.95	24.72	1.23	4.51	1.42	–0.83	−0.58	133.9	141.9
*QGZn.E1.2014–2016_Q2 (QZn 3.1)*	3	101	IPES 0166	Xpsmp 2249	14.0	5.86	1.74	4.11	6.2	1.92	4.28	1.26	–1.5	2.65	−1.15	98	102
*QGZn.E1.2014–2016_Q3 (QZn 3.2)*	3	121	IPES 0142	IPES 0180	2.1	11.97	8.44	3.53	14.39	9.53	4.86	2.8	1.36	–2.83	1.47	108	124
*QGFe.E2.2014–2016_Q1(QFe2.1)*	2	138.9	Ppmsb 001	Ppmsb 002	32.8	23.61	23.57	0.05	17.98	17.95	0.02	4.22	–0.09	0.2	−0.11	133.9	141.9
*QGFe.E2.2014–2016_Q2 (QFe3.1)*	3	121	IPES 0142	IPES 0180	2.1	21.32	21.26	0.06	16.26	16.25	0.01	4.02	–0.03	0.13	−0.1	114	124
*QGFe.E2.2014–2016_Q3 (QFe5.1)*	5	21.3	IPES 0157	IPES 0093	1.4	24.8	24.61	0.19	18.83	18.77	0.06	4.32	0.06	–0.32	0.26	19.3	32.3
*QGFe.E2.2014–2016_Q4 (QFe 7.1)*	7	24.9	IPES 0206	IPES 0015	12.3	22.02	21.97	0.05	16.61	16.61	0	4.08	–0.08	0.03	0.06	21.9	27.9
*QGZn.E2.2014–2016_Q1(QZn 2.1)*	2	138.9	Ppmsb 001	Ppmsb 002	32.8	24.89	24.58	0.31	21.88	21.77	0.11	5.05	–0.46	0.39	0.07	135.9	139.9
*QGZn.E2.2014–2016_Q2 (QZn 3.1)*	3	121	IPES 0142	IPES 0180	2.1	18.86	18.39	0.46	16.07	16.05	0.02	4.34	–0.07	0.22	−0.14	106	124
*QGFe.E3.2014–2016_Q1(QFe 1.1)*	1	452.4	IPES 0101	IPES 0139	29.1	3.94	3.93	0.01	2.92	2.91	0	–1.56	–0.06	0.09	−0.03	435.4	461.4
*QGFe.E3.2014–2016_Q2 (QFe2.1)*	2	138.9	Ppmsb 001	Ppmsb 002	32.8	20.63	20.61	0.01	15.98	15.98	0.01	3.54	0	–0.08	0.08	133.9	141.9
*QGFe.E3.2014–2016_Q3 (QFe2.2)*	2	644.9	IPES 0027	IPES 0236	23.7	3.93	3.92	0.01	2.85	2.85	0.01	–1.55	–0.06	0.09	−0.03	633.9	657.9
*QGFe.E3.2014–2016_Q4 (QFe 3.1)*	3	105	IPES 0142	IPES 0180	2.1	19.26	19.23	0.03	14.8	14.8	0	3.41	–0.02	0.01	0.01	104	112
*QGFe.E3.2014–2016_Q5 (QFe 5.1)*	5	21.3	IPES 0157	IPES 0093	1.4	21.76	21.73	0.02	16.85	16.85	0	3.64	–0.01	–0.03	0.03	19.3	34.3
*QGFe.E3.2014–2016_Q6 (QFe 7.1)*	7	24.9	IPES 0206	IPES 0015	12.3	18.97	18.96	0.01	14.56	14.55	0.01	3.39	0.08	–0.13	0.05	21.9	27.9
*QGZn.E3.2014–2016_Q1 (QZn 2.1)*	2	140.9	Ppmsb 002	IPES 0181	45.1	11.94	10.95	0.99	11.18	10.23	0.95	3.98	0.68	–1.7	1.01	139.9	143.9
*QGZn.E3.2014–2016_Q2 (QZn 3.1)*	3	105	IPES 0142	IPES 0180	2.1	25.42	24.39	1.02	22.26	21.08	1.19	5.71	–1	1.92	−0.92	104	122
*QGZn.E3.2014–2016_Q3 (QZn 6.1)*	6	478	IPES 0141	IPES 0151	27.4	3.71	3.55	0.16	2.93	2.87	0.06	–2.19	–0.32	–0.12	0.45	471	485

*LOD, overall LOD score of the QTL; LOD (A), score of the main additive effect; LOD (AbyE), score of the Genotype × Year interaction; PVE, overall proportion of phenotypic variance explained by the QTL in percentage; PVE (A), proportion of the phenotypic variance explained by additive effect at the current scanning position; PVE (AbyE), eProportion of the phenotypic variance explained by the QTL due to Genotype × Year interaction; Add, main additive effect; AbyE_01, AbyE_02, and AbyE_03, estimated additive effect of QTL at the current scanning position for each environment (Delhi, Dharwad, and Jodhpur); LeftCI and Right CI, confidence interval calculated by one-LOD drop from the estimated QTL position.*

For GZn, one common QTL was found in E1, E2, and E3 on LG 3 at significant threshold LOD value of 3.5 which was flanked by IPES0142–IPES0180 with total phenotypic variance of 14.39, 16.07, and 22.26% at E1, E2, and E3, respectively. And one more common QTL was also found at E1 and E2 on LG 2, flanked by Ppmsb001–Ppmsb002 having total PVE of 25.95 and 21.88%, respectively. Whereas, at E3, one QTL with flanked markers Ppmsb002–IPES0181was mapped on LG 2 with comparatively low total PVE of 11.18%. Two more un-repetitive QTLs between IPES0166–PSMP2249 (PVE 6.2%) on LG 3 and IPES0141–IPES0151 (PVE 2.93%) on LG 6 were detected in E1 and E3, respectively.

The proportion of the total phenotypic variance (PVE) explained by the QTLs for GFe ranged from 14.99 to 19.66%, 16.26*–*18.83, and 2.85–16.85% in E1, E2, and E3, respectively (Table 8). On the other hand, the PVE of the QTLs for GZn in E1, E2, and E3 ranged from 6.2 to 25.95%; 16.07–21.88 and 2.93*–*22.26% (Table 8). The QTLs that had the highest PVE for GFe in E1, E2, and E3 were 19.66% (IPES0157–IPES0093), 18.83% (IPES0157–IPES0093), and 16.85% (IPES0157–IPES0093), respectively. For GZn, the QTLs with the highest PVE were 25.95% (Ppmsb001–Ppmsb002), 21.88% (Ppmsb001–Ppmsb002), and 22.26% (IPES0142–IPES0180) at E1, E2, and E3, respectively.

We localized two genomic regions with pleiotropic effects on LG 2 and LG 3 for GFe and GZn at E1, E2, and E3. The first genomic region was *QGFe.E1.2014–2016_Q1* to *QGZn.E1.2014–2016_Q1* at E1, *QGFe.E2.2014–2016_Q1* to *QGZn.E2.2014–2016_Q1* at E2 and *QGFe.E3.2014–2016_Q2* to *QGZn.E3.2014–2016_Q1* at E3 linked to marker Ppmsb1–Ppmsb2 on LG 2 (Table 8). The second region was associated with *QGFe.E1.2014–2016_Q2* to *QGZn.E1.2014–2016_Q3*, *QGFe.E2.2014–2016_Q2* to *QGZn.E2.2014–2016_Q2*, and *QGFe.E3.2014–2016_Q4* to *QGZn.E3.2014–2016_Q2* linked to marker IPES0142–IPES0180, across the years, at all the three locations.

The *QGFe.E1.2014–2016_Q3* region linked to marker IPES0157–IPES0093 having largest PVE for GFe (19.66%) on LG 5 over the locations, which was also displayed 3.6 additive effect of QTL (Table 8). Whereas, the QTL for GZn having largest PVE (25.95%) was *QGZn.E1.2014–2016_Q1* on LG 2 over the locations (Table 8). Similarly, one more QTL for grain Zn (*QGZn.E2.2014–2016_Q1*) was also found with high PVE 21.88% on LG 2 at E2. The additive effect was detected for all the QTLs on which the alleles from PPMI 683 (donor parent) had positive additive effect.

### Validation of Identified QTL in Diverse Set of Germplasm

Eleven high (PPMI 708, PPMI 1102, PPMFeZMP 199, PPMI 1278, 841B, PPMI 1232, PPMI 1107, PPMI 1225, PPMI 1089, PPMWGI 146, and PPMI 1231), nine low (PPMI 1011, PPMI 759, J 2467, PPMI 1087, PPMFeZMP 47, PPMFeZMP 34, J2405, and H77/833-2, 5540B) and four checks (ICMB 98222, ICMB 93222, ICMB 07999, and ICMB 92111) for grain iron expressing pearl millet genotypes (Table 9) were selected and their DNA was used for amplification with ten consistently associated primers, IPES0142, IPES0180, IPES 0160, IPES0093, IPES0206, IPES0015, IPES0166, PSMP2249, Ppmsb001, and Ppmsb002. The amplicons of these genotypes were obtained by PCR amplification of these primers. The similar regions of reference sequence obtained were thus compared with the amplified sequences of high and low grain iron of expressing pearl millet genotypes. These markers were compared with reference genome Tift_23_D_2_B_1._

**TABLE 9:** List of pearl millet genotypes used to validate the flanked markers of QTLs for grain iron and zinc content.

**S. No**	**Genotype**	**Fe (mg/kg)**	**Zn (mg/kg)**
1	PPMI 708	114.57	73.68
2	PPMI 1102	111.64	69.77
3	PPMFeZMP 199	110.15	65.95
4	PPMI 1278	101.02	43.50
5	841B	95.86	49.79
6	PPMI 1232	92.44	69.33
7	PPMI 1107	87.31	58.36
8	PPMI 1225	85.03	49.9
9	PPMI 1089	82.66	45.64
10	PPMWGI 146	80.35	32.89
11	PPMI 1231	79.92	35.08
12	PPMI 1011	40.64	40.92
13	PPMI 759	39.98	34.41
14	J2467	39.38	34.93
15	PPMI 1087	39.22	40.26
16	PPMFeZMP 47	38.7	40.46
17	PPMFeZMP 34	36.31	45.10
18	J2405	34.71	37.48
19	H77/833-2	34.03	38.2
20	5540B	28.96	26.62
21	ICMB 98222	98	51.43
22	ICMB 93222	82	61.5
23	ICMB 07999	37	45.12
24	ICMB 92111	40	30

### *In-silico* Identification of Candidate Gene

Candidate genes identified for each QTL was shown in Table 10. In the study, we identified ten metal transporter genes. Out of the ten genes seven were identified in the genome of *Seteria italica* whereas remaining three genes were identified in the genome of *Zea mays*. Among the genes from the *Seteria italica*, Ferritin-1 was associated with the QTL *QFe 2.1* and *QZn 2.1*, Aluminum-activated malate transporter 5 and Potassium transporter 25 was identified with *QFe 3.1* and *QZn 3.1*, Probable magnesium transporter NIPA4, Metal transporter Nramp3 isoform X2, Probable cadmium/zinc-transporting ATPase HMA1, chloroplastic isoform X2 and Cadmium/zinc-transporting ATPase HMA2 were linked with *QFe 5.1*. However, among the genes from the *Zea mays*, Potassium transporter 3 was associated with *QFe 5.1* and *QFe 7.1*.

**TABLE 10:** Identification of putative candidate gene within QTL involved in enhancing Fe/Zn content.

**S. No**	**QTL**	**Gene**	**Swiss sequence ID**	**Organism**	**Identities**	**Expect score**	**Positive**
1	*QFe 2.1, QZn 2.1*	Ferritin-1, chloroplastic	K3Y9N0	*Setaria italica*	220/249 (88%)	3e-153	223/249 (89%)
2	*QFe 3.1, QZn 3.1*	Aluminum-activated malate transporter 5	K3Z099	*Setaria italica*	439/471 (93%)	0.0	447/471 (94%)
3		Potassium transporter 25	K3YQ36	*Setaria italica*	613/793 (77%)	0.0	630/793 (79%)
4	*QFe 5.1*	Probable magnesium transporter NIPA4	K3Z7C3	*Setaria italica*	76/77 (99%)	8e-40	77/77 (100%)
5		Metal transporter Nramp3 isoform X2	A0A368R0C3	*Setaria italica*	352/366 (96%)	0.0	353/366 (96%)
6		Potassium transporter 3	A0A1D6N226	*Zea mays*	188/345 (54%)	9e-120	234/345 (67%)
7		Probable cadmium/zinc-transporting ATPase HMA1, chloroplastic isoform X2	XP_012701051.1	*Setaria italica*	606/722 (84%)	0.0	626/722 (86%)
8		Cadmium/zinc-transporting ATPase HMA2	K3XUY1	*Setaria italica*	1,020/1,104 (92%)	0.0	1,042/1,104 (94%)
9		Potassium transporter 3	A0A1D6N226	*Zea mays*	298/520 (57%)	0.0	351/520 (67%)
10	*QFe 7.1*	Potassium transporter 3	A0A1D6N226	*Zea mays*		0.0	466/537 (86%)

## Discussion

### Variance Components and Heritability

Pearl millet has a great variation of mineral and agronomic traits. For both traits, the parents of this RIL population exhibited statistically significant divergent phenotypes. The BLUP means of RILs were significant for Fe and Zn content. A very wide range of variation was detected among the population for grain Fe and Zn content (Table 3).

Wider range of variation existed in the present mapping population for grain yield per plant (18.89–36.70 g) and thousand seed weight (6.84–11.79 g). Wide range of variation was earlier reported by many workers in pearl millet for yield and micronutrient traits (Govindaraj et al., 2011; Sankar et al., 2013; Vinodhana et al., 2013; Bashir et al., 2014; Sabiel et al., 2014; Anuradha et al., 2017). Greater amount of variation in the present population show that there is a lot of scope for selection of better lines to increase the grain yield in pearl millet.

The operational heritability (*H*^2^) estimates were very high and the genotypic components of variance (σ^2^*g*) for both traits were significant. Similar study was also done by Kumar et al. (2016, 2018). The trait heritability information is helpful to choose the selection procedure to be followed to improve the trait. Broad-sense heritability was high enough to allow effective QTL mapping, with moderately high to high proportions of genetic variance for the traits investigated. Higher estimates of heritability with genetic advance as a percent of mean were observed, suggesting the presence of additive gene action efficacy of selection for both traits. The traits which expressed high heritability and low genetic advance showed non additive gene action, hence heterosis breeding would be recommended for these traits. Sumathi et al. (2010) and Govindaraj et al. (2011) were also found similar result.

### Frequency Distributions

The frequency distributions of the overall means for grain iron and zinc content in the mapping population are represented by histogram (Figure 1). Likewise, previously Kumar et al. (2016, 2018) were also concluded the normal frequency distribution for both traits in pearl millet. The normal Frequency distribution for both traits was also observed in other crop like wheat (Crespo-Herrera et al., 2017). Both traits in the RIL population have phenotypic normal distributions and transgressive segregations, indicating polygenic inheritance. The presence of transgressive segregation also suggests genetic recombination (Falconer, 1996), which means that both favorable and unfavorable alleles for the traits are dispersed between the parents.

### Correlation Analysis

The understanding of the association between nutritional and agronomic traits will support the breeders to select suitable selection/breeding program for genetic improvement of associated traits such as mineral content. Several crops have been studied to determine the correlation between grain Fe and Zn contents. Iron and zinc didn’t record any significant association with grain yield and thousand seed weight albeit they in turn are correlated. In many previous studies there was negative association of Fe and Zn with yield (Kanatti et al., 2014), which hampers the effective selection of all these traits. This study is in accordance with Anuradha et al. (2017), where they didn’t notice any associations. Hence, there is a possibility for selection of high Fe and Zn content along with high yield. In current study, positive correlation between iron and zinc was observed as also reported in many previous studies (Rai et al., 2012; Govindaraj et al., 2013; Kanatti et al., 2014; Kumar et al., 2016, 2018; Anuradha et al., 2017, 2018; Velu et al., 2017). This could be due to common molecular mechanisms that regulate mineral uptake and metabolism in grains, or to common transporters controlling mineral movement within plants (Vreugdenhil et al., 2004; Ghandilyan et al., 2006; Magallanes-López et al., 2017). The strong association between these minerals in populations may be due to the co-segregation of genes for these traits. The direction and intensity of the association suggest that co-transferring superior alleles regulating these traits into the genetic backgrounds of elite lines could provide good opportunities for simultaneous genetic improvement of both micronutrients (Allouis et al., 2001). Genotypic correlations were found to be higher for all traits, implying that there is less interaction between the genetic makeup of the traits and environment.

### Linkage Map

Linkage mapping is essential for QTL mapping and marker-assisted breeding programs. The most preferred mapping population is RIL, but the accuracy of the mapping resolution is dependent on population size. The linkage maps have been predominantly constructed using F_2_ populations and up to certain extent RILs in pearl millet (Ferreira et al., 2006). Only few previous studies were done on linkage map and QTLs in pearl millet like Supriya et al. (2011) had constructed the linkage map based on 321 loci (258 DArTs and 63 SSRs) for 140 RILs. Similarly, Kumar et al. (2016, 2018) had also used DArTs and SSRs for construction of linkage map and used a smaller number of SSRs for genotyping of RILs and map development. In our study, we found 151 polymorphic SSRs out of 366 and used for linkage map development for 210 RILs. The mapping population size and number of SSRs was high compared to the Supriya et al. (2011) and Kumar et al. (2016, 2018). The present study recorded lesser polymorphism for SSRs compared to that of Senthilvel et al. (2008) who reported 74%. It may be because the parents used here for the development of RILs are closely related than that of Senthilvel et al. (2008). The distribution of SSRs across the linkage group was uniform in the current study. In terms of SSR marker positioning on the genetic map, all SSRs mapped on the similar linkage group (LG) as previously reported pearl millet maps by various researchers (Yadav et al., 2004; Senthilvel et al., 2008; Supriya et al., 2011; Kumar et al., 2016, 2018). One hundred fifty-one polymorphic SSR markers were assigned to seven previously established pearl millet linkage groups (Figure 2). The present map had a larger length than the one reported by Yadav et al. (2004); Supriya et al. (2011) and Kumar et al. (2016, 2018).

Recombinant inbred lines usually exhibit segregation distortion, because, many recessive lethal genes become homozygous during the RIL development process (Kumar et al., 2016). Segregation distortion was found in 31.57% of the loci on the current linkage map. Whereas Kumar et al. (2018) found 60% segregation distortion of DArts and SSRs. Pollen abortion is most common in pearl millet than abnormalities in female gametes, leading to a comparatively greater loss of male alleles and the subsequent skewness toward the female parent (Kumar et al., 2018). Distortion from expected Mendelian segregation has been observed previously in maize (Wendel et al., 1987; Lu et al., 2002), barley (Graner et al., 1991; Devaux et al., 1995), rice (Causse et al., 1994; Xu et al., 1997), wheat (Blanco et al., 2004; Quarrie et al., 2005), and pearl millet (Supriya et al., 2011). The protogynous nature of pearl millet also contributes to segregation distortion (Liu et al., 1994). According to Cloutier et al. (1997) and Livingstone et al. (1999), residual heterozygosity and inbreeding depression during inbred development may also contribute to segregation distortion. The residual heterozygosity in some RIL may be advantageous at the same time, because deleterious genetic combinations in the form of reduced fitness or lethality can be avoided. The segregation of nearly all loci of LG 3 was distorted in comparison to earlier reports, which may explain part of the increase in LG 3 map length.

The presence of large gaps between the centromere and telomere is a characteristic feature of pearl millet linkage maps. Senthilvel et al. (2008) found a large gap in LG 4. The previously built framework map for the cross ICMB 841-P3 × 863B-P2 had big gaps in LG 2 and LG 7 (Senthilvel et al., 2008; Yadav et al., 2011). This new population also revealed a large gap (>25 cM) in LG 1, LG 2, LG 4, and LG 6, possibly due to extreme recombination localization at the ends of LG. The interval distance between the markers in LG 4 was high suggesting additional polymorphic markers are needed to fill-up the gap. According to many researchers like Devos et al. (2000); Varshney et al. (2006), and Senthilvel et al. (2008), large gaps in the distal regions reflect regions of high recombination, rather than a lack of markers in these regions. However, these linkage groups are still incomplete, and need the addition of new markers that are located on the distal regions of the linkage groups. The number of large gaps has tried to decrease in the present study, although there is still the possibility to map more markers to fill these gaps.

### Quantitative Trait Loci for Grain Iron (Fe) and Zinc (Zn) Content and Environment Interaction

The use of QTL analysis to map populations is useful not only for identifying genomic regions associated with traits of interest, but also for using the associated marker information in breeding programs to integrate particular loci in elite germplasm. The application of QTL by environment interaction with composite interval mapping was used in the present study (Li et al., 2015), it was possible to locate several genomic regions associated with GFe and GZn during the three consecutive years over three diverse locations. Our findings support other authors’ findings that GFe and GZn are quantitative traits (Kumar et al., 2016; Anuradha et al., 2017). These previous QTL studies have also mapped QTL or genomic region for GFe and GZn in different linkage group of pearl millet, including LG 3 and LG 5, with PVE 13.6 and 10.0%, respectively, for iron content (Kumar et al., 2016). on the other hand, Anuradha et al. (2017), identified different markers on different linkage groups for both high grain iron and zinc content, namely PSMP 2261 (LG 5), IPES0096 (LG 7), IPES 0180 (LG3), and Xsinramp 6 (unmapped). Kumar et al. (2016) reported the co-localization of high grain iron and zinc content alleles/QTLs in pearl millet. It may happen because starting from uptake to final deposition into grain of iron and zinc may share some common pathways (Grotz and Guerinot, 2006). In present study we found four repetitive QTL on LG 2, LG 3, LG 5, and LG 7 with PVE ranging 16.0–18.6, 14.83–16.26, 16.88–19.63, and 14.59–16.61% in E1, E2, and E3, respectively, and the largest PVE (19.63%) was displayed by *QGFe.E1.2014–2016_Q3* in LG 5. Kumar et al. (2016) has mapped one SSR marker (IPES142) on LG 3 and Anuradha et al. (2017) has mapped IPES180 on LG3. In the present study one QTL was mapped between IPES142–IPES180 on LG 3 which is in accordance with that of Kumar et al. (2016) and Anuradha et al. (2017). Further, Anuradha et al. (2017) mapped a marker ICMP 3092 on LG 7 for Fe. Similarly, we also identified one repetitive QTL between IPES0206–IPES0015 on LG 7 and interestingly the nearest marker to this QTL is ICMP 3092 as per Rajaram et al. (2013). However, the genomic regions of this study and other studies are not comparable since Kumar et al. (2016) used DArT markers while this study used SSR markers. Similarly, the mapping method used by Anuradha et al. (2017) was association mapping while it is QTL in the present study.

The genotype by environment interaction, particularly the cross-over type interactions, has significant implications for crop performance and breeding. The ideal case for pearl millet biofortification is to obtain stable pearl millet genotypes that perform well without cross-over interaction when tested in different environments or years in a particular geographical area. The analysis of the phenotypic data in our study revealed the presence of a significant *G* × *Y* interaction. QTL analysis revealed that most of the LOD scores for the additive average effect were higher than the LOD score for the interaction (Table 7), showing that QTL with higher LOD (Add) are more stable than those with higher LOD (G Y) (Li et al., 2015). The additive effect was detected for all the QTLs for grain Fe and Zn, on which the alleles from donor parent had positive additive effect. Gaoh et al. (2020) found the additive × additive gene effects had the most important effects for grain Fe content, while additive × dominance gene effects were significant for grain Zn content in pearl millet. Similarly, Garcia-Oliveira et al. (2018) and Kumar et al. (2018) reported that epistasis plays a role in regulating grain iron and zinc content in pearl millet and cereals, respectively. Lu et al. (2008) reported 28 genome-wide additive × additive interactions for mineral elements in rice. Anuradha et al. (2012) also reported epistatic interactions between loci on chromosomes 1 and 5 for grain Zn concentration. Jin et al. (2013) reported the additive effect for Zn and partial dominant for Fe in maize.

## Validation of Identified QTLs in a Diverse Set of Germplasm

Validation of marker trait associations can reveal their worthiness for further utilization in breeding. Hence, DNA from eleven high, nine low grain iron and four check (two for high and two for low) expressing genotypes were amplified with ten SSR markers which were consistently associated with both the traits. Flanked SSR markers of identified QTLs were compared with the reference Tift_23_D_2_B_1_ genome.

### *In silico* Identification of Candidate Gene

The availability of the pearl millet genome sequence has allowed researchers to search for candidate genes involved in Fe and Zn accumulation in grains. The presence of candidate genes was investigated in genomic regions encompassing all QTLs contributing to Fe and Zn in pearl millet. QTLs identified for Fe and Zn content using composite interval mapping were analyzed *In-silico* for the presence of putative candidate genes. In the study, we identified ten metal related genes within the obtained QTLs.

Foods with higher iron content can be produced using modern genetic and molecular technologies. To increase the amount of iron and zinc in wheat, ferritin is over-expressed (Liu et al., 2016). Other mineral concentrations were also affected by ferritin expression, as reported by several studies (Drakakaki et al., 2005; Qu et al., 2005). The promoter selected to control ferritin expression is critical to iron accumulation in specific tissues. The Fe and Zn concentrations in transgenic indica rice grain improved by endosperm-specific glutelin gene promoter of soybean ferritin (Vasconcelos et al., 2003). According to Liu et al. (2016), over-expression of sickle alfalfa ferritin, which is controlled by the seed-storage protein glutelin GluB-1 gene promoter, increases grain Fe and Zn concentrations but also affects mineral homeostasis in transgenic wheat grain. Wheat TaALMT1 (ALMT, for Al-activated Malate Transporter) encoding a malate transporter involved in Al tolerance (Raman et al., 2005). Over expression of TaALMT1 in wheat, barley, and tobacco-cell suspension increases the efflux of Al-activated malate and increases the tolerance to Al stress (Pereira et al., 2010). Root exudation of organic acid such as malate/citrate may play an important role in providing iron to plants (Brown, 1983). Citrate efflux is crucial for iron translocation, and this is mediated by the efflux transporter FRD3 in Arabidopsis (Green and Rogers, 2004) and its orthologue FRDL1 in rice (Yokosho et al., 2016). Barak and Chen (1984) reported a positive effect of K on iron absorption which was associated with acidification of the rhizosphere. Sakaguchi et al. (1999), observed secretion of mugineic acid family phytosiderophores (MAs) from the use of potassium gradient, and content of potassium in barley roots increased with iron-deficiency. Grotz et al. (1998) identified the zinc transporter genes in *Arabidopsis thaliana.* Yang et al. (2020) studied on activity of zinc transporter (OsZIP9) mediates zinc uptake in rice. Plants acquire Mg from the environment and distribute in the ionic form via Mg^2+^-permeable transporters. In Arabidopsis, MGT6, (plasma membrane-localized magnesium transporter) mediates Mg^2+^ uptake under Mg-limited conditions (Yan et al., 2018). The Magnesium/proton exchanger protein is a vacuolar exchanger of protons with cytosolic Mg^2+^ and Zn^2+^ in Arabidopsis. Hence, it may be possible that Mg and Zn share a common pathway of transport in plants.

#### Ferritin Gene (Ppmsb001–Ppmsb002)

Plant ferritins are important iron storage proteins that serve as both an acceptor and a donor of iron in metabolic processes (Briat et al., 2010). It shares structurally and functionally similarities with animal ferritins. Plant ferritin is generally observed in cells which are inactive in photosynthesis such as seed, root, root nodules, and others. Plant ferritins are nuclear encoded and located primarily in plastids (Zielińska-Dawidziak, 2015). This protein also controls the cellular concentration of transition metals, metal ions apart from iron in the mineral core (including cadmium, beryllium, zinc, aluminum, and lead) (Kumar and Prasad, 1999). Many gene families like FER, ZIP, NRAMP, YSL, and NAS, were involved for mineral translocation from soil to the grain filling site. These gene families have been identified in plants such as *Arabidopsis thaliana* (Weber et al., 2004), *Setaria italica* (Alagarasan et al., 2017), and *Oryza sativa* (Neeraja et al., 2018). In the current report, the ferritin gene was reported by *in silico* method, which is similar to the Ferritin-like (*PglFER*1) gene family previously reported by Mahendrakar et al. (2020). With reference to previous studies it is clear that ferritin gene regulates the iron metabolism in plants.

#### Al^3+^ Transporter in Plants (IPES0142–IPES0180)

Aluminum is a potentially phytotoxic metal and Al tolerant plants excrete organic acids such as malate, citrate, and oxalate from root tips, depending on the plant (Ryan et al., 2011). The organic acid exudation is vital for plants tolerating metal and nutritional stress at the root-soil interface. Aluminum interferes the uptake or transport of nutrients such as Ca, B, Fe, Mn, P, Mg, and Cu or K (Keltjens and Tan, 1993; Keltjens, 1995; Lukaszewski and Blevins, 1996; Ślaski et al., 1996). Root elongation and nutrient (B, Fe, Mg, Ca, and P) absorption may also be inhibited by Al or low pH soils. Root exudation of organic acid such as malate/citrate may play a key role in supplying Fe to plants (Brown, 1983). Hence, with the consideration of previous studies, we assumed that the aluminum activated malate transporter gene is responsible to cease the aluminum absorption by plant root and also induce the iron absorption.

#### K^+^ Transporters in Plants (IPES0142–IPES0180, IPES0157–IPES0093, and IPES0206–IPES0015)

Oertli and Opoku (1974), as well as Barak and Chen (1984), identified a positive effect of K on Fe nutrition, which was linked to rhizosphere acidification (excessive K^+^ uptake and consequent release of H^+^ ions by roots to maintain a cation/anion balance). The activity of H+ ATPase pumps located at the plasma membrane is responsible for rhizosphere acidification, which is essential for nutrient acquisition. The liberation of proton is in the favor of uptake of Fe and other macro and micronutrients, especially under the deficiency conditions (Houmani et al., 2015). Hughes et al. (1992) found a significant effect of K^+^ on proton efflux and ferric reduction mechanisms (Strategy I of iron uptaking by roots). They also identified that K^+^ play specific physiological functions in the biosynthetic pathway of mugineic acid production and in the transport of Fe^3+^- phytosiderophore complex (Strategy II). So, we can assume that K^+^ transporter also regulate the iron absorption in plants under iron stress condition.

#### Zn^2+^ Transporters in Plants (XIPES0157–XIPES0093)

In present study, we have notified one zinc transporter gene of ZIP superfamily that was earlier reported in pearl millet by Mahendrakar et al. (2020). In higher plants, members of the ZIP family are involved in metal uptake, transport, and accumulation of iron and zinc in plant cells. The first identified zinc transporter genes were ZIP1, ZIP2, and ZIP3 in *Arabidopsis thaliana* (Grotz et al., 1998). Several ZIPs, including OsIRT1, OsIRT2, OsZIP1, OsZIP3, OsZIP4, OsZIP5, OsZIP7, and OsZIP8, have been identified as being responsible for Zn uptake from soil, translocation within the root and from the root to the shoot, and storage in rice seeds (Yang et al., 2009; Lee et al., 2010).

#### Mg^2+^ Transporters in Plants (IPES0157–IPES0093)

Mg^2+^ transporters are used to maintain Mg in plant organs. Among the proteins potentially involved in Mg^2+^ transport, Magnesium transporter (MGT) family Mg^2+^ transporters plant was the most well-investigated protein (Kobayashi and Tanoi, 2015). Meanwhile, the other transporters of Mg^2+^ are also possible such as Non-selective cation channels (NSCCs) (Guo et al., 2003). According to Demidchik and Maathuis (2007), Voltage-independent NSCC (VI-NSCC) reported the uptake of several cations including Mg^2+^, Ca^2+^, and Zn^2+^ at the resting membrane potentials. The Arabidopsis Magnesium/proton exchanger (MHX) protein is a vacuolar exchanger of protons with cytosolic Mg^2+^ and Zn^2+^. Hence, it may be possible that Mg and Zn share a common pathway of transport in plants.

We have identified one of the gene in ferritin gene family that was already reported in pearl millet via *insilico* studies and validated by qRT-PCR gene expression (Mahendrakar et al., 2020). The transporter genes like Al, Mg, and K transporter are reporting for the first time in pearl millet. Whereas these transporter gene family were found previously in many different crops such as Al transporters gene in wheat (Li T. et al., 2017) and Rice (Sonoda et al., 2003); Mg transporter gene in Maize (Li et al., 2016, 2018) and Arabidopsis (Li H. et al., 2017); K transporter gene in Maize (Zhang et al., 2012) and Barley (Santa-María et al., 1997); and Zn transporter gene in Arabidopsis (Grotz et al., 1998), rice (Ishimaru et al., 2005; Lee et al., 2010; Neeraja et al., 2018) and Maize (Li et al., 2013).

## Conclusion

Enhancing the level of grain Fe and Zn content among elite cultivars of pearl millet become the priority area in answering the nutritional imbalance among poor inhabitance of the semi-arid tropics, especially women and children. Unravelling the genetic principles involved in inheritance of complex traits like grain micronutrient content in pearl millet via, trait-specific mapping enables rapid genetic gains. Huge genetic variation among RIL population observed under the present study can be further used to develop high yielding micronutrient rich cultivar through heterosis breeding. The two co-localized QTLs on LG 2 and LG 3, for both the minerals found to promising as it could simultaneously transferred to the parental lines of elite pearl millet hybrids through marker-assisted breeding programs.

## Data Availability Statement

The datasets presented in this study can be found in online repositories. The names of the repository/repositories and accession number(s) can be found in the article/Supplementary Material.

## Author Contributions

CS, SPS, and AK designed and supervised the overall research and contributed in the preparation of the manuscript. CS and SPS provided technical guidance for creating liaison among multi-environment locations, and edited the manuscript for final submission. TS, SMS, JB, and MM executed the field experiments. TS and SMS performed the phenotyping, carried out statistical analysis, and prepared the manuscript draft. CB and NA reviewed the manuscript. All authors contributed to the article and approved the submitted version.

## Conflict of Interest

The authors declare that the research was conducted in the absence of any commercial or financial relationships that could be construed as a potential conflict of interest.

## Footnotes

1 https://blast.ncbi.nlm.nih.gov/

2 http://www.softberry.com/berry.phtml?topic=fgenesh&group=programs&subgroup=gfind

## Supplementary Material

Supplementary Figure 1 A to D shown the QTLs for grain Fe content on LG 2,3,5, and 7 at Delhi location.

Supplementary Figure 2 A and B shown the QTLs for grain Zn content at LG 2 and 3 at Delhi location.

Supplementary Figure 3 A to D shown the QTLs for grain Fe content on LG 2,3,5, and 7 at Dharwad location.

Supplementary Figure 4 A and B shown the QTLs for grain Zn content at LG 2 and 3 at Dharwad location.

Supplementary Figure 5 A to D shown the QTLs for grain Fe content on LG 2,3,5, and 7 at Jodhpur location.

Supplementary Figure 6 A and B shown the QTLs for grain Zn content at LG 2 and 3 at Jodhpur location.
