# Supplementary figures and images for: Multi-Environment Quantitative Trait Loci Mapping for Grain Iron and Zinc Content Using Bi-parental Recombinant Inbred Line Mapping Population in Pearl Millet
Source: Front Plant Sci. 2021 May 18;12:659789. doi: 10.3389/fpls.2021.659789 (PMC8169987; doi:10.3389/fpls.2021.659789)

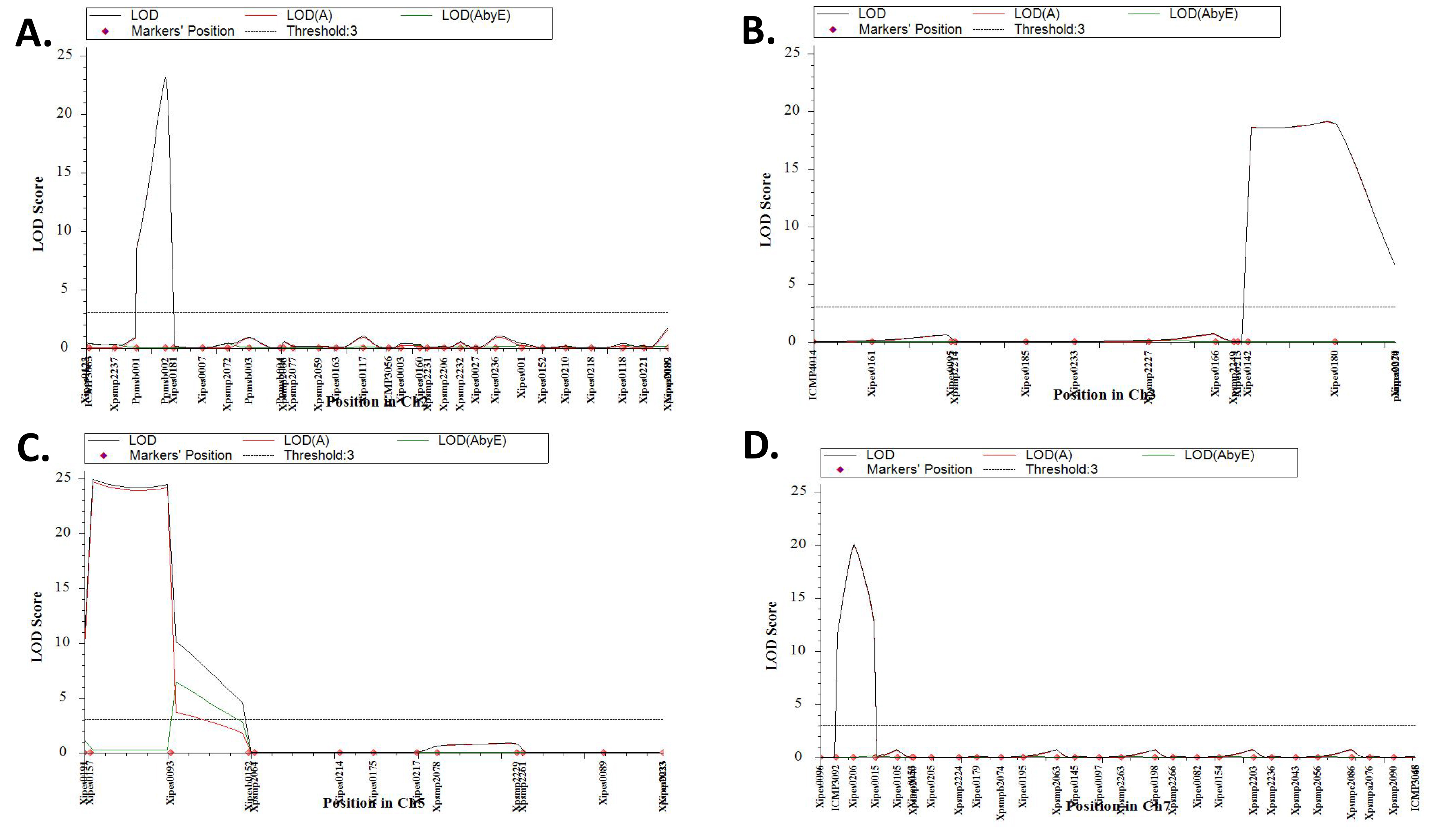

Supplement: Supplementary Figure 1 — A to D shown the QTLs for grain Fe content on LG 2,3,5, and 7 at Delhi location. [file Image_1.JPEG]

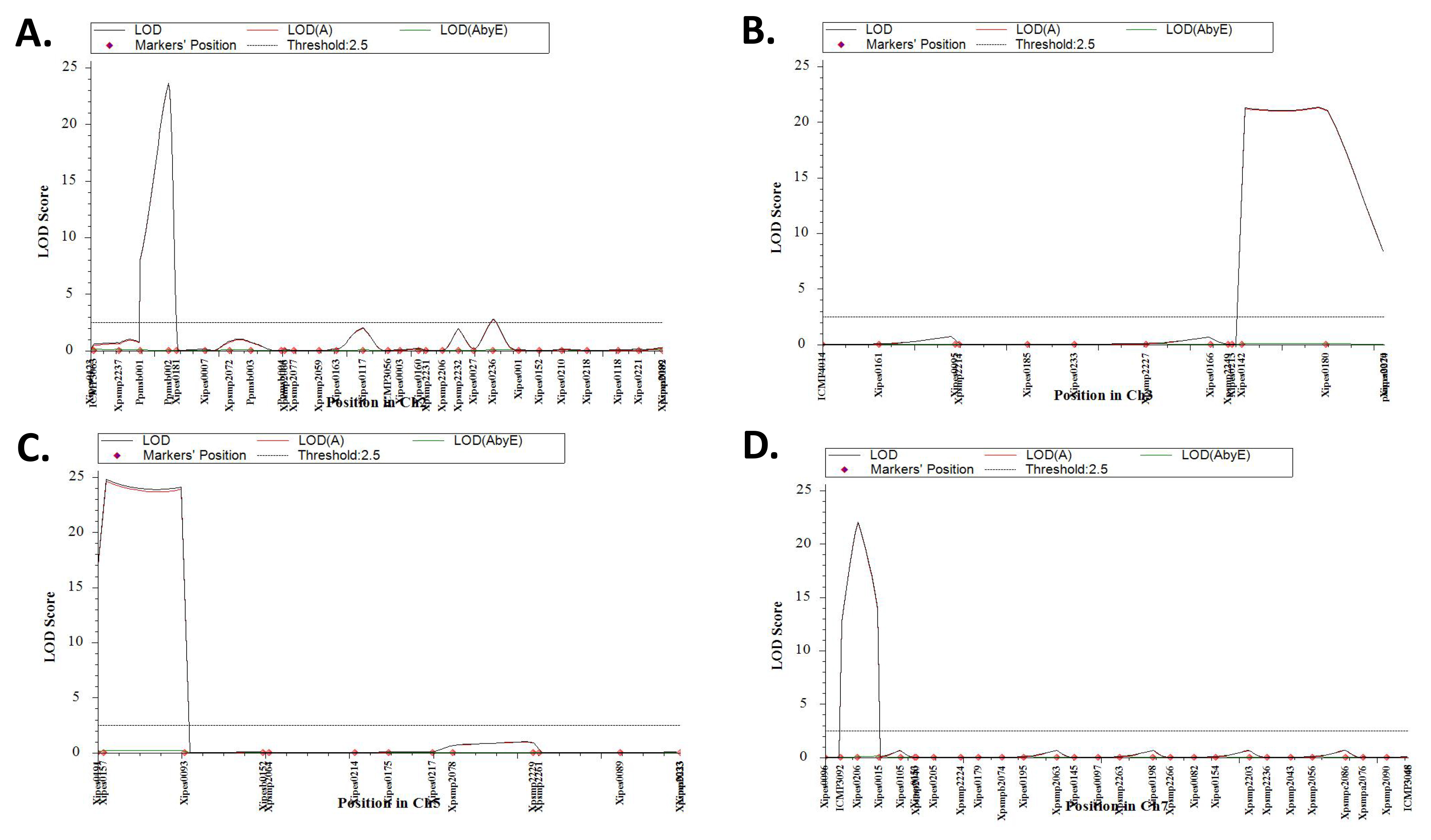

Supplement: Supplementary Figure 2 — A and B shown the QTLs for grain Zn content at LG 2 and 3 at Delhi location. [file Image_2.JPEG]

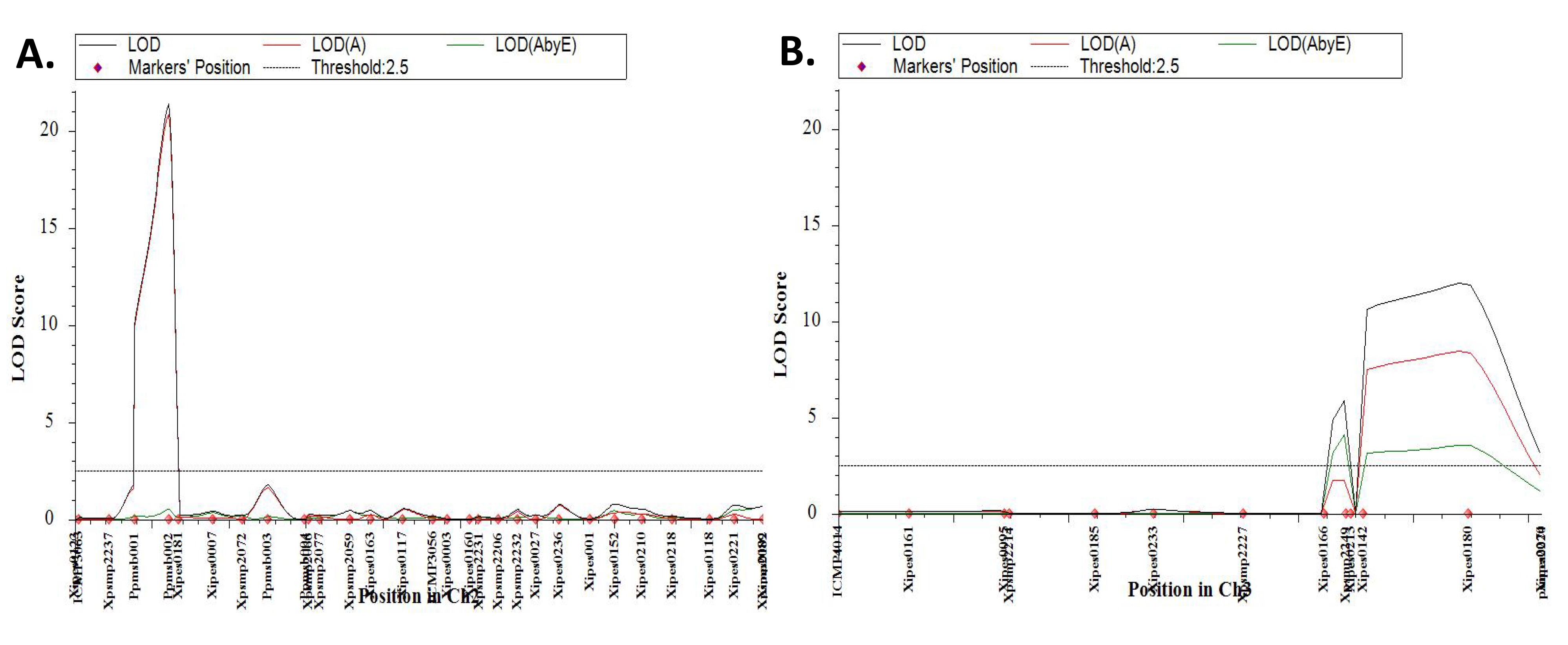

Supplement: Supplementary Figure 3 — A to D shown the QTLs for grain Fe content on LG 2,3,5, and 7 at Dharwad location. [file Image_3.JPEG]

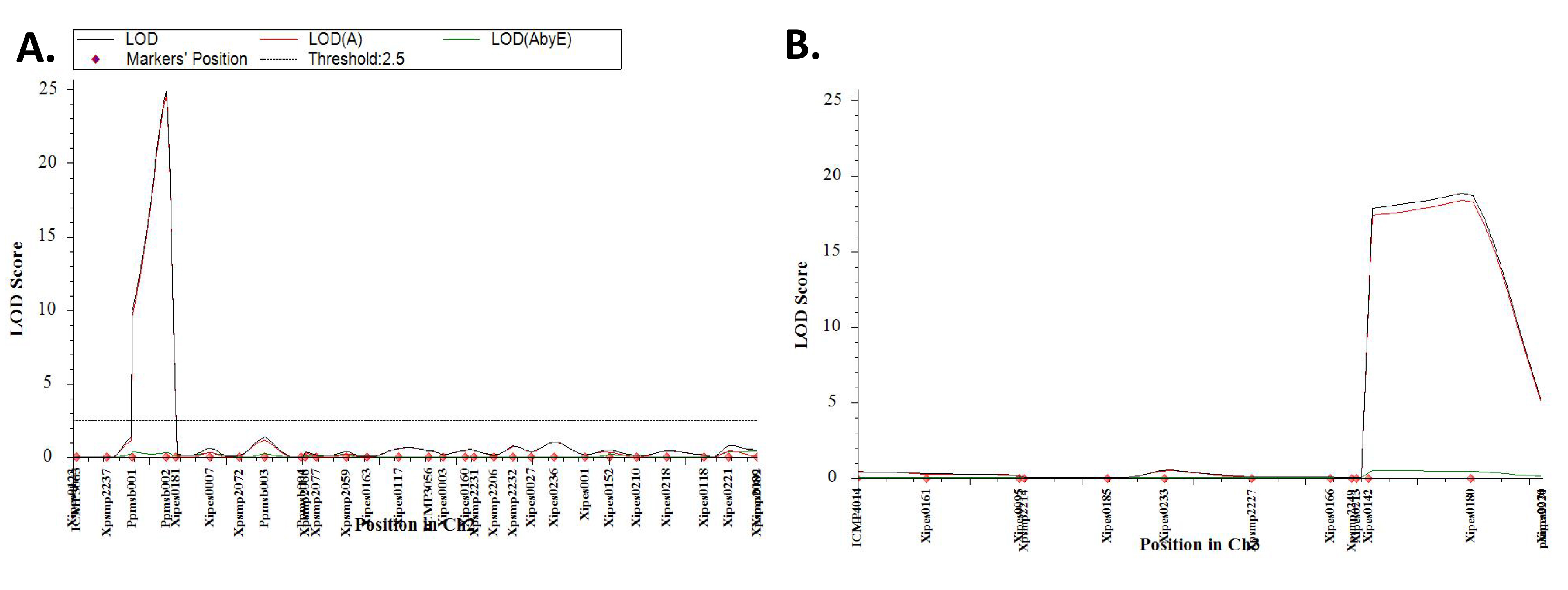

Supplement: Supplementary Figure 4 — A and B shown the QTLs for grain Zn content at LG 2 and 3 at Dharwad location. [file Image_4.JPEG]

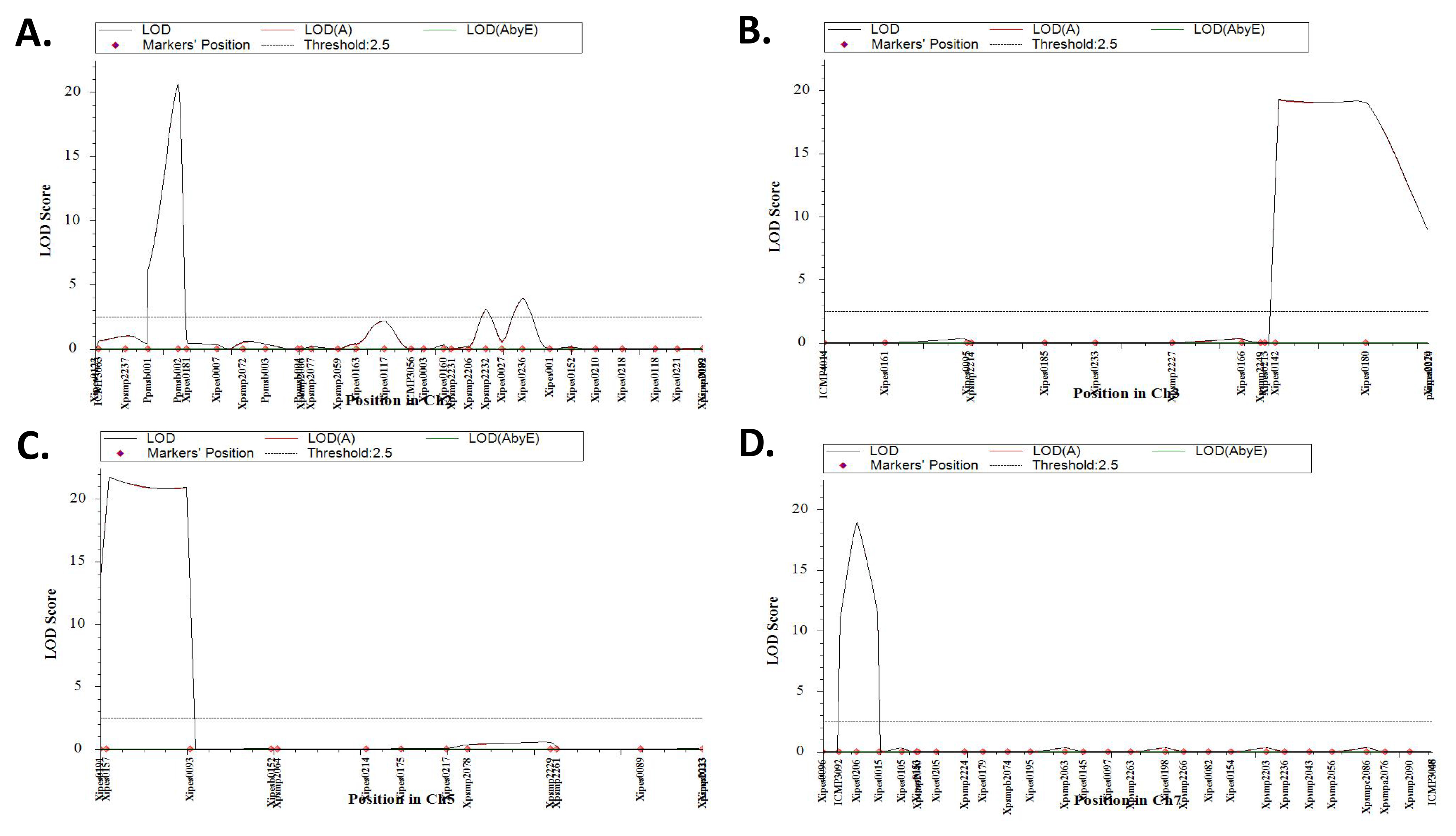

Supplement: Supplementary Figure 5 — A to D shown the QTLs for grain Fe content on LG 2,3,5, and 7 at Jodhpur location. [file Image_5.JPEG]

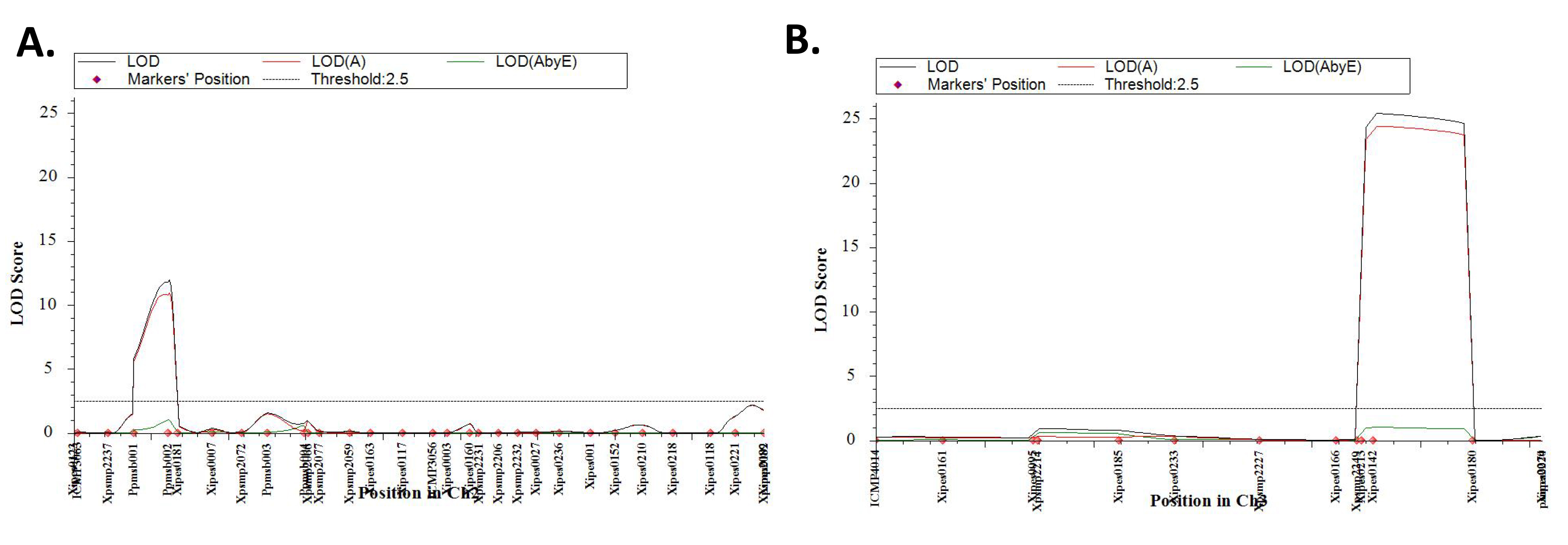

Supplement: Supplementary Figure 6 — A and B shown the QTLs for grain Zn content at LG 2 and 3 at Jodhpur location. [file Image_6.JPEG]
